# Expression of Small RNA in *Aphis gossypii* and Its Potential Role in the Resistance Interaction with Melon

**DOI:** 10.1371/journal.pone.0048579

**Published:** 2012-11-16

**Authors:** Sampurna Sattar, Charles Addo-Quaye, Yan Song, James A. Anstead, Ramanjulu Sunkar, Gary A. Thompson

**Affiliations:** 1 College of Agricultural Sciences, The Pennsylvania State University, University Park, Pennsylvania, United States of America; 2 The Schatz Center for Tree Molecular Genetics, Department of Ecosystem Science and Management, The Pennsylvania State University, University Park, Pennsylvania, United States of America; 3 Bioinformatics Core Facility, Oklahoma State University, Stillwater, Oklahoma, United States of America; 4 Department of Biochemistry and Molecular Biology, Oklahoma State University, Stillwater, Oklahoma, United States of America; Natural Resources Canada, Canada

## Abstract

**Background:**

The regulatory role of small RNAs (sRNAs) in various biological processes is an active area of investigation; however, there has been limited information available on the role of sRNAs in plant-insect interactions. This study was designed to identify sRNAs in cotton-melon aphid (*Aphis gossypii*) during the *Vat*-mediated resistance interaction with melon (*Cucumis melo*).

**Methodology/Principal Findings:**

The role of miRNAs was investigated in response to aphid herbivory, during both resistant and susceptible interactions. sRNA libraries made from *A. gossypii* tissues feeding on *Vat^+^* and *Vat^−^* plants revealed an unexpected abundance of 27 nt long sRNA sequences in the aphids feeding on *Vat^+^* plants. Eighty-one conserved microRNAs (miRNAs), twelve aphid-specific miRNAs, and nine novel candidate miRNAs were also identified. Plant miRNAs found in the aphid libraries were most likely ingested during phloem feeding. The presence of novel miRNAs was verified by qPCR experiments in both resistant *Vat^+^* and susceptible *Vat^−^* interactions. The comparative analyses revealed that novel miRNAs were differentially regulated during the resistant and susceptible interactions. Gene targets predicted for the miRNAs identified in this study by *in silico* analyses revealed their involvement in morphogenesis and anatomical structure determination, signal transduction pathways, cell differentiation and catabolic processes.

**Conclusion/Significance:**

In this study, conserved and novel miRNAs were reported in *A. gossypii*. Deep sequencing data showed differences in the abundance of miRNAs and piRNA-like sequences in *A. gossypii*. Quantitative RT-PCR revealed that *A. gossypii* miRNAs were differentially regulated during resistant and susceptible interactions. Aphids can also ingest plant miRNAs during phloem feeding that are stable in the insect.

## Introduction

Gene regulation at the post-transcriptional level has garnered considerable attention in recent years due to an increased understanding of the regulatory roles of small RNAs (sRNA). The first regulatory sRNA was discovered in *Caenorhabditis elegans* almost twenty years ago [1], and since then, progress in deep sequencing technologies combined with sophisticated bioinformatic analyses have facilitated the identification of a large number of sRNAs. Three main groups of animal sRNAs: microRNAs (miRNAs), endogenous small interfering RNAs (endo-siRNA) and Piwi-interacting RNAs (piRNAs) have been classified, based on their distinctive characteristics, biogenesis processes, and association with Argonaute proteins [2]. Among these three classes, miRNAs are the most studied and best understood. The biogenesis of miRNAs involves the action of two RNase III proteins, Drosha and Dicer, that are required for processing the hairpin shaped structures to 22–23 nucleotide (nt) long mature miRNAs. The 21 nt endo-siRNAs in insects and mammals are produced in a RNA-dependent RNA polymerase (RdRP) independent manner, requiring a Dicer-dependent process [2]. Endo-siRNAs mainly originate from transposon transcripts, intergenic repetitive elements, and endo-siRNA cluster loci. piRNAs also originate from intergenic repetitive elements, including retro-transposons, but do not require Dicer for processing. piRNAs, originally reported from *Drosophila melanogaster* germ cells [3], are easily distinguished from the other two classes due their longer size (24–29 nt). This group of sRNA interacts with Piwi proteins and silences selfish genetic elements contributing towards germ line stability [4], [5]. Recently piRNAs from neural cells were shown to play a role in the epigenetic control of memory related synaptic plasticity [6].

Traditionally most of the well characterized insect sRNAs were restricted to miRNAs sequences from *Drosophila* spp.; however with the recent availability of RNA-seq and whole genome sequencing data, miRNA sequences from other insect species such as silkworm (*Bombyx mori*), honeybee (*Apis melifera*), mosquito (*Anopheles gambiae*), locust (*Locusta migratoria*), beetle (*Tribolium castaneum*), pea aphid (*Acyrthosiphon pisum*), German cockroach (*Blatella germanica*) and brown planthopper (*Nilaparvata lugens*) have been deposited in miRBase [7], [8]. Interestingly, the orders Diptera (*Drosophila spp.* and *A. gambiae*) and Lepidoptera (*B. mori*) contribute most of the insect miRNA sequences in miRBase. The depth of sRNA sequence information from the other insect orders is still limited. Pea aphid and brown planthopper are the only Hemipterans represented in miRBase [9], [10] and *A. pisum* is the only aphid species to have miRNA sequences reported [9]. The order Hemiptera includes harmful agricultural insect pests such as aphids, whiteflies, scales, and hoppers. Many of the insects belonging to the order Hemiptera possess specialized mouth parts capable of piercing plant tissues and feeding directly from the vasculature. Piercing-sucking insects not only deplete photoassimilates, but also transmit pathogenic plant viruses. Hemipterans are hemimetabolus as they do not undergo complete metamorphosis like other insect groups. Aphids are typically parthenogenetic during most part of their life cycle and give live birth to genetically identical nymphs. The unique feeding habit combined with the ability to rapidly reproduce makes these insects some of the most damaging pests of economically important crops worldwide.

*Aphis gossypii* (cotton-melon aphid) is a destructive insect pest on a wide range of economically important host plants. Chemical control measures for *A. gossypii* are becoming limited as this species developed resistance to a wide variety of insecticide classes [11]. Therefore, significant research effort has focused on identifying and developing host-plant resistance. Resistance to *A. gossypii* imparted by the *Vat* (virus aphid transmission) gene has been identified in several geographic *Cucumis melo* plant introductions and introgressed into commercial melon lines [12], [13], [14], [15], [16]. The *Vat* gene is a member of CC-NBS-LRR family of plant resistance (*R*) genes, which confers dual resistance to *A. gossypii* and non-persistent viruses transmitted by this aphid [12], [17]. Resistance against *A. gossypii* exhibits two possibly overlapping, modes of action; antixenosis (non-preference of the aphids) and antibiosis (reduction in aphid performance and fecundity) [18]. The antixenotic component of the resistance trait is characterized by delays in sustained phloem sap ingestion on resistant plants due to rapid interruption in feeding after the stylets penetrate the phloem [19]. The antibiotic component extends the pre-reproductive period of the aphid and shortens both the reproductive and post-reproductive periods resulting in fewer progeny [19]. The overall life span of an individual aphid is reduced and after the final molt, aphids feeding on the resistant *Vat^+^* plants are smaller in size than those feeding on the susceptible *Vat^−^* melon plants. Thus, important reproductive, developmental, and morphological changes occur in aphids in response to *Vat*-mediated resistance. In this study, a survey of sRNAs from *A. gossypii* feeding on *Vat^+^* and *Vat^−^* melon plants was conducted to better understand the role of these regulatory, non-coding RNAs in the developmental changes that occur in response to the *Vat*- mediated resistance mechanism.

## Results

### Sequence analysis of the aphid libraries

Two *A. gossypii* sRNA libraries were generated from aphids collected after 48 hours of feeding on *Vat^+^* (*Vat^+^* aphid library) and *Vat^−^* (*Vat^−^* aphid library) melon plants, respectively. The libraries were sequenced using Illumina GAII analyzer and the library data is available at NCBI Gene Expression Omnibus (GEO) under the series GSE38641. Illumina sorts the sequence data in a “Tag_Count” file format, where each sequence read (tag) is provided with the number of times (counts) it appeared in the library. The sRNA sequencing data in the tag count files have been used for in depth analyses.

**Figure 1:**
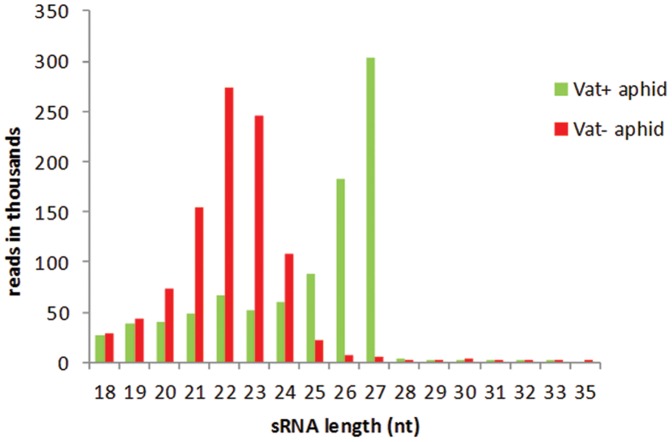
Size distribution of sRNA sequences in *Vat^+^* and *Vat^−^* aphid libraries.

**Figure 2:**
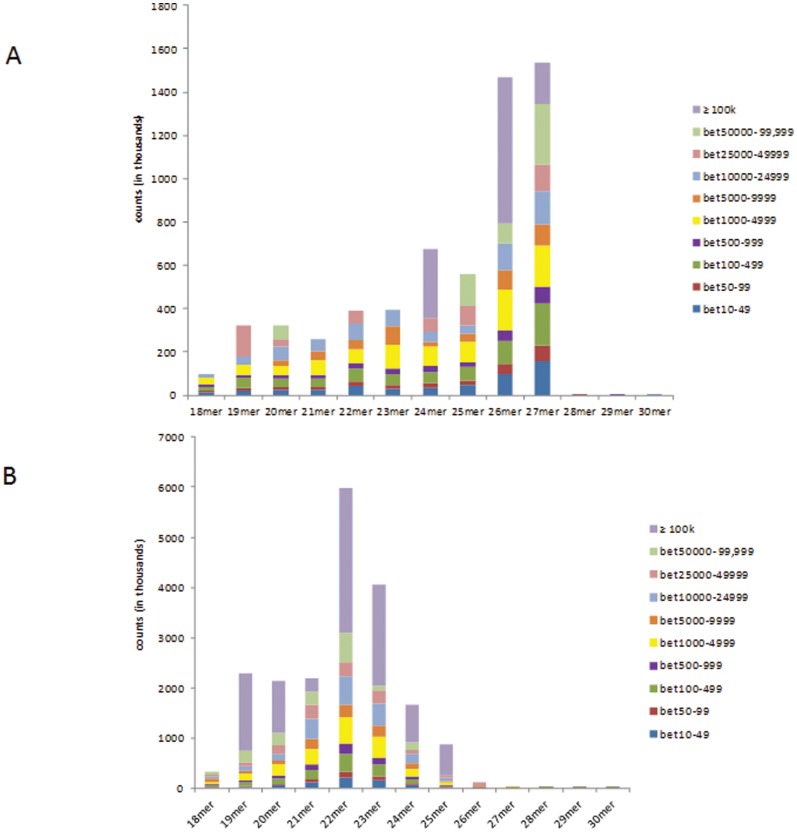
Sequence abundance complexity in the *Vat^+^* and *Vat^−^* aphid libraries in the 18–30 nt size range. (A) *Vat^+^* aphid library (B) *Vat^−^* aphid library.

The *Vat^+^* aphid library generated approximately 2.5 million reads, and after adapter trimming and removing redundant sequences, the number of usable sequences was 918,621. Similarly, for the *Vat^−^* aphid library approximately 1.2 million reads were obtained and the usable number of sequences was 974,781. Before the libraries were searched for putative miRNA sequences, rRNAs and tRNAs were discarded and the number of usable sequences after this step was 32,668 and 45,898 for *Vat^+^* and *Vat^−^* aphid libraries, respectively (Table 1). Reads with counts of 10 or more in the size range of 18–30 nt were retained and designated as distinct reads (File S1). The distribution of sRNAs was strikingly different in two aphid libraries. The size distribution pattern of the reads revealed that *Vat^+^* aphid library was over-represented by 26–27 nt sequences, whereas the *Vat^−^* aphid library was dominated by 22 nt sequences (Figure 1). Both the libraries were further analyzed to determine their count complexity (Figure 2). In the *Vat^+^* aphid library, reads in the 26 nt size category showed very high abundance, with two reads accounting for ≥100,000 counts each, comprising 45% of that size category, thereby making this size category the most prominent in the *Vat^+^* aphid library (Figure 2A). In the 27 nt size category; a single read accounted for over 100,000 counts (12.5%), four reads contributed to counts in the range of 50,000–99,999 each (18%) and another four reads recorded counts in the range of 25,000–49,999 each (8%) (Figure 2A). The *Vat^−^* aphid library showed a bias towards 22 nt sequences (Figure 1); based on the count distribution seven reads recorded counts of ≥100,000 each (48%) and eight reads contributed to counts in the range of 50,000–99,999 each (10%) (Figure 2B). The 23 nt size category in the *Vat^−^* aphid library was represented by six reads with counts ≥100,000 each (50%), one read in the 50,000–99,999 count category (2.3%), and seven reads in the 25,000–49,999 count category (6.7%). The number of reads in each count category for both the libraries is listed in File S2. Thus, it is evident that the *Vat^−^* aphid library is abundant in 22 nt sequences and the *Vat^+^* library is enriched in longer sRNA sequences of 26–27 nt.

**Table 1:** Aphid sRNA library statistics.

	*Vat^+^* aphid library	*Vat^−^* aphid library
Raw reads	2520036	1211182
Adaptors removed	918621	974781
rRNA/tRNA matches removed	32668	45898
Match to miRNAs (miRBase)	545	1073
Match to miRNAs from *A.pisum*	170	336
Match to *A. gossypii* EST	4837	2206
Match to *A. pisum* genome	12618	17159
Match to *M. persicae* EST sequences	7256	4049

### Identification of conserved miRNA in aphid libraries

Conserved *A. gossypii* miRNAs were identified by searching the sub-set of sRNAs, designated as distinct reads, against the repository of animal miRNAs in miRBase (Release 18, http://www.mirbase.org/). During homology searches, two mismatches were allowed, and 81 miRNA sequences belonging to 56 miRNA families were identified (Table 2) from both libraries combined. As observed from the size distribution counts, the number of 22 nt sequences was noticeably lower in the *Vat^+^* aphid library, which was reflected by the number of miRNA sequences identified in that library. In comparison, to the 77 miRNA sequences identified in the *Vat^−^* aphid library, only 69 miRNA sequences were reported from the *Vat^+^* aphid library (Table 2). Several miRNA families including miR-100, miR-137, miR-307, miR-316, miR-3041 and miR-3042 were absent in *Vat^+^* aphid library. Certain miRNAs like miR-133a, miR-1357, miR-29b and miR-87b were not found in *Vat^−^* aphid library. Interestingly, some miRNA families (miR-10, miR-184, miR-2, miR-276, miR-87, miR-9 and let-7) were represented by fewer members in the *Vat^+^* aphid library, whereas in *Vat^−^* aphid library these families were strongly represented by more members. The abundance of these miRNA sequences in each library is reported in ‘transcripts per million’ (TPM). The TPM profile represents a measure of miRNA expression levels [20], and the results show different patterns of accumulation for the conserved miRNAs between the two libraries (Table 2). A general trend towards lower miRNA accumulation was observed in *Vat^+^* aphid library. However, some miRNA families like miR-1357, miR-1692, miR-184, miR-310, miR-996 and miR-998 showed enhanced accumulation in *Vat^−^* aphid library, as suggested by the library counts (Table 2). Library counts have been useful to survey expression profiles for further investigation [10], [21]. In addition to conserved miRNA sequences, miRNA* sequences were also identified for miR-276 and miR-1 from both the libraries.

**Table 2:** Conserved miRNA counts in TPM^a^.

miRNA	Sequence	*Vat^+^* aphid library	*Vat^−^* aphid library
miR-1	AAATGTAAAGAAGTATGGAG	219138	373212
miR-1-2-as	TCCACACTTCTTTACATTCCA	77	95
miR-10	CAAATTCGGTTCTAGAGAGGTTT	14220	21167
miR-10a	GACCCTGTAGATCCGAATTTGTA	1067	72241
miR-10b	TACCCTGTAGACCCGAATTTGT	0	21
miR-10c	CACCCTGTAGATCCGAATTTGT	1073	5395
miR-100	GACCCGTAGATCCGAACTTGTGT	0	90
miR-124	TAAGGCACGCGGTGAATGCCATT	150	710
miR-13	TATCACAGCCGTTTTTGACAATT	307	3027
miR-133	TTGGTCCCCTTCAACCAGCTGT	0	145
miR-133a	TTGGTCCCCTTCAACCAGCTGT	98	0
miR-1357	ATTATGAGATCTGAGGGCA	21	0
miR-137	TATTGCTTGAGAATACACGTAG	0	55
miR-14	TCAGTCTTTTTCTCTCTCCTAT	144	445
miR-1692	CGTAGCTCAGATGGTAGAG	32	22
miR-182	CTTGGCACTGGAAGAATTCACAG	31	241
miR-183	AATGGCACTGAAAGAATTCACGG	1485	18035
miR-184	AGGACGGAGAACTGATAAAGGC	45622	271424
miR-184a	AGACGGAGAACTGATAAGGGC	34155	721
miR-184b	TGGACGGAGAACTGAAAGGGC	23596	158
miR-190	AGATATGTTTGATATTCTTGGTTG	59	210
miR-2a	GATCACAGCCAGCTTTGATGAGC	1009	1375
miR-2a-1a	CACAGCCAGCTTTGATGAGCA	2381	13849
miR-2a-1b	TATCACAGCCAGCTTTGTATGAGCG	978	1580
miR-2b	TCACAGCCAGCCTTGATGAGCA	101	13
miR-2c	TATCACAGCCAGCTTTGATGAGT	0	69
miR-200	CAATACTGTCAGGTAATGATGT	0	83
miR-210	CTTGTGCGTGTGACAGCGGCTAT	225	1402
miR-228	AATGGCACTAAAAGAATTCACGGG	13192	23062
miR-2138	AAGGGAACGGGCTTGGCAGAAT	229	1644
miR-236	TAATACTGTCAGGTAATGATGT	168	905
miR-252	CTAAGTACTAGTGCCGCGGGAG	303	1455
api-miR-252b	CTAAGTAGTAGCGCCAACGGTGA	1839	38118
miR-263a	AATGGCACTGAAAGAATTCAC	23	39
miR-263b	CTTGGCACTGGAAGAATTCACAGA	324	418
miR-275	ACAGGTACCTGAAGTAGCGCGT	420	16485
miR-276	AGCGAGGTATAGAGTTCCTACG	4743	42621
miR-276a	AGGAACTTCATACCGTGCTCT	4465	6790
miR-277	TAAATGCACTATCTGGTACGACA	64	1047
miR-278	TCGGTGGGACTTTCGTTCGTTT	145	768
miR-279	TGACTAGATCCACACTCATCC	85	405
api-mir-2796	GTAGGCCGGCGGAAACTACTAG	34	361
miR-29	TAGCACCATTTGAAACCAGTAT	0	23
miR-29b	TAGCACCATTTGAAACCAGT	11	0
miR-310	TATTGCACATGTCCCGGCCA	47	13
miR-34	TGGCAGTGTGATTAGCTGGTTGT	223	858
miR-305	ATTGTACTTCATCAGGTGCTCT	310	829
api-miR306	TCAGGTACCAAGTGATTTCTGA	1664	28783
miR-307	CCACAACCTCCTTGAGTGAGCGA	0	40
miR-315	TTTTGATTGTTGCTCAGAAAGCC	496	3811
miR-316	TGTCTTTTTCCGCTTTGCTGCCG	0	774
miR-317	GGAACACAGCTGGTGGTATCTCA	627	3112
miR-375	TTTGTTCGTTCGGCTCGAGTC	2220	6045
api-mir-3041	GTAAAGCTTTGATGACGGGATA	0	988
mir-3042	GAGGGCAGATTATTTCTGATAC	0	39
api-miR3049	TCGGGAAGGCAGCTGCGGCGGACT	27	1236
api-mir-3050	TGAGATCTTGATAAACTCGC	949	7143
miR-7	TGGAAGACTAGTGATTTTGTTGT	254	681
miR-71	TGAAAGACATGGGTAGTGAGAA	125	11331
miR-71c	TGAAAGACATGGGTAGTGAGAT	0	4279
miR-750	CCAGATCTAACTCTTCCAGCTGA	63	3276
miR-8	AATACTGTCAGGTAATGATGTC	170	2647
miR-87	ATGAGCAAAGTTTCAGGTGTGC	1181	21711
miR-87a	GTGAGCAAAGTTTCAGGCTTGT	15	802
miR-87b	GTGAGCAAAGTTTCAGGCGT	12	0
miR-9	TCTTTGGTTATCTAGCTGTA	329	2348
miR-9-2	CCTTTGGTTATCTAGCTGTATGA	0	22
miR-92	TATTGCACATGTCCCGGCCAAT	0	3287
miR-92a	GATTGCACATGTCCCGGCCAAT	75	259
miR-92b	GATTGCACTTGACCCAGCCTGC	124	100
miR-92c	TATTGCACATGTCCCGGCCAAT	587	2269
miR-927	TTTAGAATTCCTACGCTTTACC	37	128
miR-993	CAAGCTCGTCTCTACAGGTATCT	658	6387
miR-996	TGACTAGAGTTACACTCGTCA	24	20
miR-998	TAGCACCATGGAATTCAGCT	502	40
miR-1000	ATATTGTCCTGTCACAGCAGTA	13	125
miR-iab-4-5p	ACGTATACTAAATGTATCCTGA	15	85
bmo-bantam	TGAGATCATTGTGAAAGCTAACT	301	3974
let-7	TGAGGTAGTTGGTTGTATAGA	1943	4652
let-7d	TGAGGTAGTTGGTTGTATAGTA	147	1338
let-7e	TGAGGTAGTTGGTTGTATAGTT	0	126

a Transcripts per million.

Twelve aphid-specific miRNAs were identified in the aphid libraries (Table 3). Till date, aphid miRNAs have been reported only from *A. pisum* [9]. The TPM profile revealed that eight out of the twelve aphid- specific miRNAs were expressed in *Vat^+^* aphid library (Table 3). Ap-miR-X15, Ap-miR-X43, Ap-miR-X71, and Ap-miR-X81 were not reported from the *Vat^+^* aphid library (Table 3).

**Table 3:** Aphid-specific miRNA counts in TPM^a^.

miRNA	Sequence	*Vat^+^* aphid library	*Vat^−^* aphid library
Ap-miR-X12	CTTGGTAACACATACGTCTTTAG	13	142
Ap-miR-X15	CCGGACATTGTAAGAACGGCCC	0	45
Ap-miR-X25	TCTTTGGGATTTAATAGAGCCGGT	391	957
Ap-miR-X26	TGTGACTGTACTTTTCATGGATGGGA	102	10
Ap-miR-X31	CCAGTCTTGCATTTATTCCACT	171	605
Ap-miR-X36	TGTTAGTATAACTCTTAGTAAC	15	45
Ap-miR-X43	TGGGGTTTCAATAGGCATTTACC	0	139
Ap-miR-X47	CAGCCGGTGGTGACTGTTTCCATA	19	138
Ap-miR-X57	CAAAACATTCAAAATTCCCTGC	17	78
Ap-miR-X59	TGGTAACTCCAAACCATTGCCGG	94	1906
Ap-miR-X71	CCACGGTTGAACAAGGTACCATA	0	10
Ap-miR-X81	CGGACATTGTAAGAACGGCCC	0	32

a Transcripts per million.

### Identification of novel *A. gossypii* miRNAs

**Figure 3:**
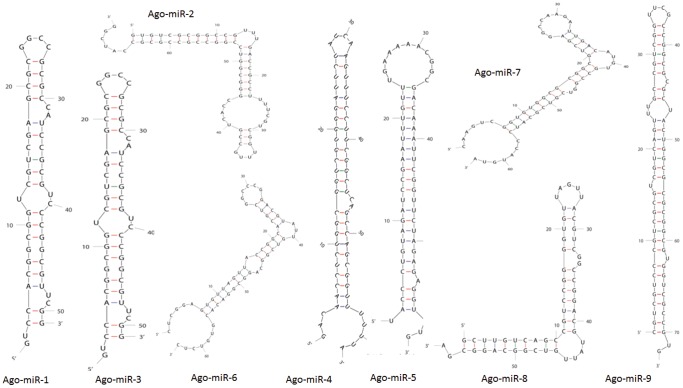
Predicted secondary structure of pre-miRNA transcripts of novel miRNAs identified in *A. gossypii*.

**Figure 4:**
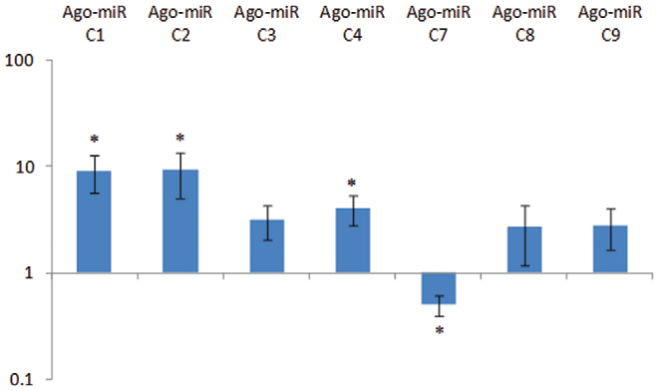
Expression profile of the candidate novel miRNAs in *A. gossypii* during a resistant interaction. Mature miRNA expression level was estimated by quantitative real time PCR using RNA isolated from aphids feeding on a *Vat^+^* and *Vat^−^* plants for 48 h. The expression levels were normalized using eF1α as an internal reference gene. The fold changes were calculated using Pfaffl's method and represents change in the expression level of miRNA relative to aphid on *Vat^−^* plant as control. Data are averages of seven independent replicates ± standard error of mean. Significance between *Vat^+^* and *Vat^−^* aphid was determined by student's t-test, and is represented by * (p≤0.05).

Sequences in both the libraries were analyzed for the presence of novel *A. gossypii* miRNAs. *A. gossypii* EST sequences (http://www.aphidbase.com/aphidbase/downloads) and the genome sequence of *A. pisum* (http://www.aphidbase.com/aphidbase/downloads) were used by the miRDeep algorithm to map the precursor sequences in order to identify novel miRNAs. The miRDeep algorithm uses the probabilistic model of the miRNA biogenesis to score the compatibility and the position of the sequence sRNA to the secondary structure of the miRNA precursor [22]. Because animal miRNAs have a small precursor length, 100 bp regions were folded into stem-loop structures to identify the putative novel miRNA sequences (Figure 3). Nine candidate novel miRNAs were identified from *A. gossypii* (Table 4). Stem-loop quantitative real-time PCR (qPCR) was performed to validate the expression of these candidate novel miRNAs. Seven out the nine yielded PCR products that could be detected (Figure 4). Ago-miR-C1, Ago-miR-C2 and Ago-miR-C4 were significantly (p≤0.05) up-regulated in *Vat^+^* aphid library. Ago-miR-C3 was also up-regulated, with a p-value of 0.051 that was just below the significance threshold. Ago-miR-C7 was the only novel miRNA in the *Vat^+^* aphid library to show significant down-regulation (p≤0.001).

**Table 4:** Novel miRNA sequences.

miRNA	Mature miRNA sequence	Precursor miRNA sequence
ago-miR-C1	GUCCACGGCGGUCGUCGAGCGC	GUCCACGGCGGUCGUCGAGCGCGGCCGCGCCAUCCGCGUCCCGGCGUUCGG
ago-miR-C2	GGGCGGUCCGGCCGCCGCGCCAUCGG	GUGUCGCGGCCGUUUGACCGCCUUUUCGUCGGUUUGCCGUCACCGGGCGGUCCGGCCGCCGCGCCAUCGG
ago-miR-C3	GUCCACGGCGGUCGUCGAGCGCGGC	GUCCACGGCGGUCGUCGAGCGCGGCCGCGCCAUCCGCGUCCCGGCGUUCGG
ago-miR-C4	ACAACCUCUGGCGGUCGUGGGA	GACAACCUCUGGCGGUCGTGGGAUUCUAUCAAGUUUCCUUCGGCUCAGCCAGCGGUUUUUUA
ago-miR-C5	AAAUUCGGUUCUAGAGAGGUUUGUG	UACCCUGUAGAUCCGAAUUUGUUUGAAAAACGGCGACAAAUUCGGUUCUAGAGAGGUUUG
ago-miR-C6	CUCGGAGUGUUAGUUACCGGC	CUCGGAGUGUUAGUUACCGGCACGUCGGCCCGGACGUAUUGUCGGCAGGCGGACACGUGUCUC
ago-miR-C7	CAAGUC GGUGUGGCG CGGCGU	CAAGUCGGUGUGGCGCGGCGUCGAGGCCAAGAUUGACAUGUGCCGGUCGUCGCAUCCCAUGUA
ago-miR-C8	GGACGUAUUGUCGGCAGGCGA	GCUUGUCAGCCGUCCGGGGUGUUAGUUACGUCGGCCGGACGUAUUGUCGGCAGGCGA
ago-miR-C9	CGUCGUCCCGUCGCGUCGUCAG	CGUCGUCCCGUCGCGUCGUCAGUUUGCCGUCGGUUCGCCGGCGCGCUACUGGCGCGCGGCGUGGUCGCCGUG

### Identification of plant miRNA families in the aphid libraries

**Figure 5:**
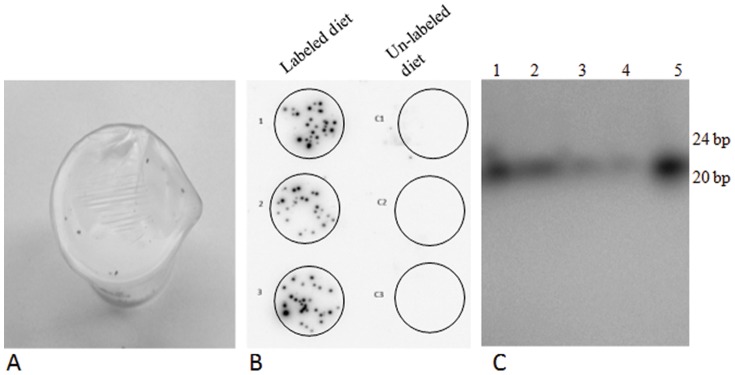
*In vitro* feeding assay with *A. gossypii*. A. Sachet feeding system. B. Honeydew droplets collected on a blotting sheet viewed by phosphor-imager C. 15% PAGE analysis of RNA and honeydew isolated from the aphids feeding on labeled diet and exposed to autoradiograph. Lane 1–2, Total RNA isolated from aphids; 3–4, honeydew elutions; 5, positive control ([γ^-32^P]-ATP labeled ds-21 nt).

Six plant miRNA families were identified from the aphid libraries; miR156/miR157, miR166, miR168, miR2911 and miR2916 (Table 5). All of these plant miRNAs were also present in melon sRNA libraries [23], [24]. Two of the six, miR166 and miR168, were identified in the *Vat^+^* aphid library. However, confirmatory experiments using stem-loop qPCR could detect only miR166, miR168 and miR2911 in both *Vat^+^* aphid and *Vat^−^* aphid tissues (data not shown). The plant derived miR156/miR157 and miR2916 did not yield detectable amplification products. To further validate if sRNAs could be transferred to aphid tissue via phloem feeding, an *in-vitro* feeding assay was performed with 5′ [γ^-32^P]-ATP labeled synthetic double stranded (ds) 21 nt oligomers. The [γ^-32^P]-ATP labeled ds-21 nt was presented to the aphids over a four day period in a liquid diet composed of 0.5 M sucrose enclosed within two parafilm layers (Figure 5A). A disc of blotting paper placed at the bottom of the container was used to collect honeydew (excreta) droplets. After four days of feeding, phosphor-imaging detected strong signals on the discs in patterns that were consistent with the distribution of honeydew (Figure 5B). RNA extracted from both aphid tissues and honeydew eluted from the discs was separated by PAGE and exposed to X-ray film revealing the [γ^-32^P]-ATP labeled synthetic double stranded (ds) 21 nt oligomers in each sample (Figure 5C).

**Table 5:** Plant miRNA families identified from aphid sRNA libraries.

Plant miRNA	*Vat^+^* aphid library*	*Vat^−^* aphid library*	Presence in phloem exudates
miR156/miR157	0	17	yes
miR166	2628	960	yes
miR168	48	38	yes
miR2911	0	40	unknown
miR2916	0	307	unknown

* counts in TPM (transcripts per million).

### Analysis of the longer (26–27 nt) sRNAs

The *Vat^+^* aphid library had a strong representation of the longer sRNA sequences (26–27 nt) (Figure 1). Remarkably, the accumulation of these 26–27 nt sequences was about 30 times higher in *Vat^+^* aphids in comparison to *Vat^−^* aphids. *In silico* experiments were performed to identify these longer sRNA sequences and to map their origin. Previous studies have shown well-characterized piRNA sequences tend to have lengths ranging between 26 and 30 bases and are often derived from repetitive elements or originate from transposons. In the absence of an assembled and annotated genome database for *A. gossypii*, homology-based searches for transposon-like origins for the subset of sRNA sequences with lengths greater than 26 nt was performed using the available transposon database of *A. pisum*. Results of homology searches from the *Vat^+^* aphid library revealed 942,169 reads out of the total 2,064,014 (46%) in 26–27 nt long read category that were mapped to transposons (data not shown). Similarly for *Vat^−^* aphid library, 51% of the reads in the 26–27 nt category originated from the transposable elements. Based on the *A. pisum* annotation, the putative repeat classes identified from the distinct sequences include Polintons, miniature inverted repeat-transposable elements (MITEs), Helitrons, terminal inverted repeats (TIRs), long terminal repeats (LTRs), long interspaces nuclear elements (LINEs), short inverted nuclear elements (SINEs) and a large diverse class of unclassified transposable elements (NoCats). An informal examination of the base composition of the sequences shows a depletion of adenosine nucleotides. A thorough exploration of the sequences using the DREME program [25] in the MEME motif finding software package (version 4.8.1) [26] discovered an overrepresentation of GC-rich motifs in both libraries with the top five motifs found in each library shown in Table 6. The longer sRNA sequences were also analyzed to determine if any of the sequences originated from the endosymbiont genome. Aphids accommodate the primary endosymbiont *Buchnera aphidicola* within specialized cells called bacteriocytes that contribute to the overall nutritional health of the aphid [27]. Homology searches allowing one mismatch with the *Buchnera* genome revealed 4.6% of the 26–27 nt sequences in the *Vat^+^* aphid library and 0.85% in the *Vat^−^* aphid library were of bacterial origin (data not shown).

**Table 6:** Overrepresented motifs in the piRNA (distinct) sequences of the *A. gossypii* libraries.

*Vat^+^* aphid library		*Vat^−^* aphid library	
Motif	P-value	Motif	P-value
GGAGCCY	6.4e-745	CCGACD	4.8e-531
GACGCAC	2.3e-346	RCCGYGGA	9.40e-305
CTHYACC	1.5e-345	GWAGA	1.10e-224
ACSGCC	1.9e-340	CGGKAGCC	4.50e-188
CTYCGGWC	1.40e-285	AYRCGTCC	2.10e-176

### Target prediction of miRNAs

*In silico* target prediction analysis was conducted to further understand the role of the different sRNA molecules identified in the aphid tissue. While several computational methods exist for predicting mRNA targets of metazoan miRNAs [28], [29], the PITA program was selected because the prediction is not based on target-site conservation, yet the program can detect species-specific miRNA targets [30]. As a control, the PITA program was used to compute miRNA targets in the well characterized *D. melanogaster* genome. All the sequences for the 304 miRNAs and 3′-UTRs from the *D. melanogaster* genome database [31], [32] were used for the control analysis. The *D. melanogaster* miRNA-target prediction results corroborated with the results from previous *D. melanogaster* miRNAs target studies (data not shown). A total of ∼2,600 targets were predicted for all the identified *A. gossypii* miRNAs, including the conserved and novel sequences (Table 7) using the *A. gossypii* Unigenes. Since a single miRNA can target more than one mRNA, the number of miRNA-target pairs was about 4,130 (Table 7). The PITA program predicted several isoforms and multiple UTRs as targets for particular miRNAs. After removing redundancy, the number of targets for *A. gossypii* conserved miRNAs identified from the *A. pisum* 3′ UTR sequences was approximately 4,500. Following similar analyses, 814 targets were predicted for the nine candidate novel miRNAs (File S5).

**Table 7:** Target prediction results for conserved and novel miRNAs from *A. gossypii* using a ΔΔG cut-off threshold of −10.0.

Database	Targets for conserved miRNA*		Targets for novel miRNA	
	microRNA targets	microRNAs -target pairs	microRNA targets	microRNAs -target pairs
*A. gossypii* (ESTs)	17,245	24,417	6,036	7,398
*A. gossypii* (Unigenes)	2,221	3,232	709	902
*A. gossypii* (Contigs)	959	1,398	252	312
*A. pisum* (3′-UTRs)	4,826	6,936	824	962
*M. persicae* (Contigs)	3,114	4,646	719	862

* Conserved miRNA includes the aphid-specific miRNAs.

**Figure 6:**
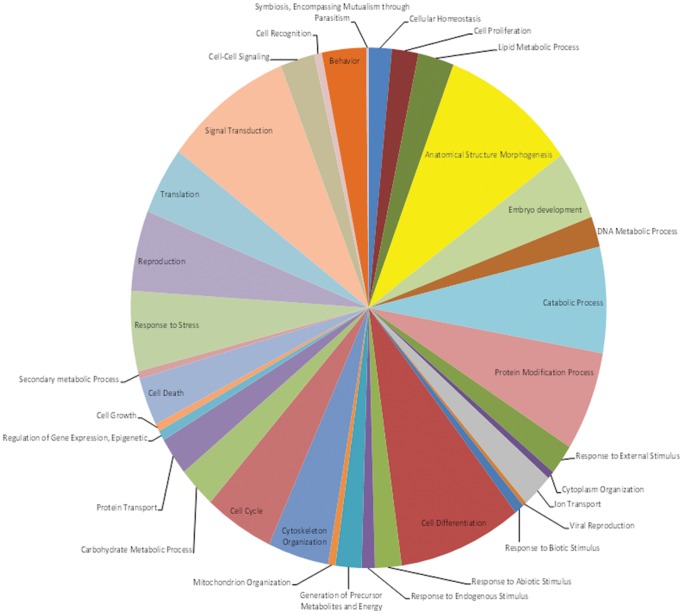
Functional categories of the PITA predicted target genes of the miRNAs identified from *A. gossypii* UniGenes by BLAST2GO analysis.

The biological functions of the predicted targets of the *A. gossypii* miRNAs were analyzed using the currently available gene ontology (GO) annotations of the *A. pisum* genes. The *A. pisum* genome database contains 46,360 GO terms for 11,440 genes. Using protein homology information, GO terms associated with all the *A. gossypii* sequences with significant hits to the *A. pisum* proteome were extracted. Table 8 summarizes the homology and annotation results for all four sequence data sets used. Similar patterns of functional annotation were observed for all the four databases (File S3) used in target predictions of the *A. gossypii* miRNAs (File S6). The largest single group of the target genes (9.2% of the total annotated GO terms) identified was annotated to have functional roles in morphogenesis and anatomical structure determination (Figure 6). Genes belonging to signal transduction pathways (8.7%), cell differentiation (8.4%), and catabolic processes (6.6%) were also frequently identified as targets.

**Table 8:** Gene Ontology annotation of PITA predicted *A. gossypii* miRNA targets.

Database	BLAST Hits	GO Hits	Number of GO Terms
*A. gossypii* (ESTs)	53,118	33,606	171,559
*A. gossypii* (Unigenes)	5,978	3,594	18,836
*A. gossypii* (Contigs)	2,616	1,722	9,608
*M. persicae* (Contigs)	7,959	4,724	24,619

The RNAhybrid program [33] was also used to obtain a more conservative set of miRNA target predictions for *A. gossypii*. GO annotations for these datasets are provided in File S7. Between 38–43% of the *A. gossypii* mRNA targets were identified in both RNAhybrid and PITA predictions when searched against *A. gossypii* ESTs and unigenes and *A. pisum* genome. The number of overlapping targets increased to 46% when using the *M. persicae* EST database with both methods. These results reflected differences in the two prediction algorithms and their cut-off threshold values and metrics.

## Discussion

The regulatory role of sRNAs in biological processes such as development, metabolism and environmental stress responses has been well documented across different taxa. In recent years, the involvement of insect miRNAs has been studied in the context of the well-defined process of metamorphosis [34], [35], [36], [37]. However, there has been limited progress in understanding the roles of miRNAs and their target genes in insect-host interactions, particularly those interactions that involve genetic host plant resistance. Host plant resistance to aphids in melon is due to the presence of the *R*-gene *Vat*. Interesting phenotypic differences were observed in aphids during the resistance interaction [38]. When aphids are forced to survive on the resistant host plant they show reduced growth, retarded development and decreased reproduction as compared to aphids thriving on the susceptible host. This study examined the molecular mechanism of aphid responses to *R* gene-mediated host plant resistance by comparing two aphid sRNA libraries generated from *A. gossypii* feeding on *Vat^+^* (resistant) and *Vat^−^* (susceptible) melon plants.

An interesting feature of the two libraries was the different size distribution patterns found in aphids feeding on susceptible or resistant plants. The *Vat^−^* aphid library generated from aphids feeding on a susceptible host plant showed an unimodal distribution that was highly represented by 22–23 nt sequences of non-coding RNA that were identified as miRNAs (Table 2). Aphids feeding on resistant plants showed a bimodal size distribution pattern of sRNAs with additional enrichment of 26–27 nt sequences (Figure 1). Unimodal size distributions with miRNAs as the most abundant size class has been observed in German roach and *Aedes* mosquito [39], [40], whereas, bimodal size distribution with abundance peaks at 20–22 nt and 27–29 nt have been reported during different developmental stages in silkworm and brown planthopper and for blood fed female *Culex quinquefasciatus* [10], [37], [40]. A shift in size distribution from unimodal to bimodal occurred between two developmental phases in migratory locust [35]. In locusts, the 22 nt peak was identified as miRNAs and the peak of longer sRNAs was due to the presence of piRNA-like sequences. Wei and co-workers (2009) [35] concluded that 26–29 nt sRNAs are involved in the process of phase changes in locust, with the development of each form (gregarious and solitary phase) showing a distinct expression pattern of specific types of sRNA. Like locusts, aphids exhibit a high degree of phentoypic plasticity and can develop different forms (alate versus apterous and sexual versus asexual). The 22 nt peak in the aphid libraries have been confirmed as miRNAs (Table 2). In the absence of *A. gossypii* genome sequence the 26–27 nt category of longer sRNAs were mapped to the repeat elements from *A. pisum*. Mapping analysis revealed that 46% of the longer sRNA from *Vat^+^* aphid library originated from repeat elements in the genome and could be classified as piRNA-like elements. The abundance of piRNA-like sequences in the *Vat^+^* aphid library raises questions about their involvement in aphid responses to *Vat*-mediated resistance.

A detailed analysis of the miRNAs expressed in the two aphid libraries also showed differences in accumulation of miRNAs between the two libraries. The proportion of miRNAs in the *Vat^−^* aphids was approximately five times greater than the *Vat^+^* aphids (Figure 1). Aphids feeding on resistant *Vat^+^* plants lacked many of the miRNAs that were expressed in the *Vat^−^* aphids (Table 2). Differences in the accumulation pattern were also observed between both the libraries for most of the conserved miRNAs; the *Vat^+^* aphid library showed a general trend towards reduced accumulation of miRNAs (Table 2). Similar variability was also observed for aphid-specific miRNAs between the *Vat^+^* and *Vat^−^* aphid libraries, where four of the twelve families were not detected in *Vat^+^* aphids (Table 3). However, some miRNAs (miR-133a, miR-1357, miR-184a, miR-184b, miR-2b, miR-310, and miR-998) showed enhanced accumulation in the *Vat^+^* aphid library, indicating their potential role in the resistance interaction. Further evaluation is required to determine the importance of the differential expression of these miRNAs and their involvement in the resistance mechanism. The expression profile of the novel miRNAs also showed differences between the libraries. Quantitative PCR analysis detected most of the novel miRNAs identified in the *A. gossypii* libraries. The expression of Ago-miRC1, Ago-miRC2 and Ago-miRC4 were significantly up-regulated in the *Vat^+^* aphids (Figure 4); however, amplification products were not detected for Ago-miRC5 and Ago-miRC6, which could be the result of limited primer specificity.

The differential accumulation of miRNAs in the two libraries suggests that these non-coding RNAs play some role in regulating aphid developmental processes in the resistance interaction. Cross-genome conservation of gene targets often aids in identifying the functionality of miRNAs. The regulatory roles of conserved miRNAs have been investigated extensively in *D. melanogaster*, *C. elegans* and mouse. Several conserved miRNAs have experimentally validated roles; miR-1 is involved in muscle growth and cardiogenesis, miR-14 in fat metabolism, miR-9 in cellularization, miR-311/miR-312 in dorsal closure and miR-315 in wing development [41], [42], [43]. The most abundant miRNA expressed in *Vat^−^* aphid library was miR-1, and its enhanced expression could induce muscle development by down-regulating myostatin [44]. Another miRNA with enhanced expression in the *Vat^−^* aphid library, miR-133, originates from the same loci as miR-1 in mouse and is transcribed in a tissue specific manner during skeletal muscle development in both mouse myoblast cells and *Xenopus laevis* embryos [45]. Activation of wing development by ectopic miR-315 in *Drosophila*, which was abundant in the *Vat^−^* library, could be an appropriate response for aphids feeding on a quality host plant as population growth results in crowding effects, which induce wing formation [43], [46]. Loss of function experiments in *Drosophila* showed miR-184 plays a role in oogenesis and egg development [47]. Differential expression of miR-184 in aphids could be indicative of changes in resource allocation from or to reproduction, particularly as aphid ovariole number has been found to be influenced by host quality [48] Recently, an important regulatory role has been demonstrated for miR-310 in neurotransmitter release in *Drosophila* [49], and this miRNA showed enhanced expression in *Vat^+^* aphid library.

*In silico* target prediction for *A. gossypii* miRNAs were performed using PITA and RNA hybrid program [30], [33]. *In silico* detection of bona fide targets of metazoan miRNAs is a challenging task because target sites lack extensive Watson-Crick base pairing and hence, the miRNA recognition elements are very short motifs [28], [50], [51]. As a result, a single metazoan miRNA gene family can be predicted to target hundreds of messages in an organism. A combination of *in silico* prediction and gene ontology annotation of the miRNA targets indicated that many of the target genes are involved in the regulation of anatomical morphogenesis and cell differentiation processes. Gomez-Orte and co-workers (2009) [34] have shown that metamorphosis is regulated by miRNA in the German cockroach. RNAi silenced expression of Dicer-1 in last instar nymphs led to the depletion of the mature miRNAs that impaired the ability of the nymphs to molt into adults. Nymphoids were morphologically similar to those resulting from juvenile hormone (JH) treatment [34]. The reduced accumulation of mature miRNAs in the *Vat^+^* aphids that target genes involved in morphogenesis and cell differentiation could be responsible for reduced growth and fecundity in aphids that are nutritionally challenged when forced to survive on resistant plants.

Another interesting feature of the aphid sRNA libraries was the detection of miRNAs of plant origin. It appears that during phloem sap ingestion members of several plant miRNA families (miR156/miR157, miR166, miR168, miR2911 and miR2916) were transferred into the aphid tissues or were present in the contents of the gut as illustrated by the *in vitro* feeding assays (Figure 5). Three of these miRNAs (miR156/miR157, miR166 and miR168) have been reported to be present in phloem sap of pumpkin (*Cucurbita maxima*), apple (*Malus domestica*), canola (*Brassica napus*) and lupin (*Lupinus sp*) [52], [53], [54], [55]. The presence of the remaining plant miRNAs (miR2911 and miR2916) in the phloem sap has not been confirmed; however, their identification in the aphid libraries suggests that these miRNAs are also present in the phloem sap of melon. The perfect match of these sequences to conserved plant miRNAs strongly indicates that they originate from the host plant. Furthermore, the possibility that large transcripts acquired from plant phloem sap by the aphid can be processed into miRNAs seems remote, as this would require the ingested plant pri-miRNA to be efficiently transferred and processed at high numbers in aphid gut cell nuclei. Interestingly, only two plant miRNA families (miR166 and miR168) were detected from *Vat^+^* aphids. This could reflect the well-documented observations that aphids on *Vat^+^* plants spend less time ingesting phloem sap than aphids feeding on *Vat^−^* plants [19], [56].

In summary, this study identified 81 conserved, 12 aphid-specific and nine novel miRNAs from *A. gossypii*. The novel miRNAs were validated by qPCR, and it was established that these miRNAs are differentially expressed in the *Vat^+^* and *Vat^−^* aphids. In general, a reduced accumulation of mature miRNAs was observed for *Vat^+^* aphids, suggesting their expression is regulated in response to the *Vat*-mediated resistance mechanism. Targets of these miRNAs were predicted using *in silico* methods, and it was observed that most of the target genes were involved in morphogenesis and anatomical structure determination, indicating that these miRNAs have regulatory roles in aphid development, growth and fecundity.

## Materials and Methods

### Plant and insect growth conditions

The nearly isogenic AR 5 (*Vat* ^+^, resistant) and PMR 5 (*Vat^−^*, susceptible) melon lines were grown in controlled conditions of 23°C, 60% relative humidity and a photoperiod of 16∶8 h (light∶dark) for 4 weeks until they reached the four leaf stage. *A. gossypii* clonal colony was reared and maintained on susceptible honeydew melon plants in a controlled growth chamber at 21°C, with a photoperiod of 16∶8 h (light∶dark).

### Insect infestations

The third and fourth leaves from four-week-old melon plants, both AR 5 and PMR 5, were used for aphid infestations. Each leaf was exposed to 50 apterous aphids confined in a 2.5 cm diameter clip cage. A mixture of aphids from different growth stages were used to mimic a natural crop infestation. Infestations were terminated after 48 hours. After this period, all aphids were gently brushed off the leaf tissue, collected in a nuclease free microfuge tube, snap frozen in liquid nitrogen and stored at −80°C. All experiments were biologically independent and at least seven independent replicates were used for each melon line.

### Cloning of sRNA and Illumina sequencing

Total RNA was isolated by TRIzol® reagent (Invitrogen) following the manufacturer's instructions for plants with high polysaccharide content, since aphid body is mostly composed of the sugary phloem. The RNA was enriched for low molecular weight (LMW) RNA by precipitation with 25% PEG and 5M NaCl [57]. The LMW RNA quality was evaluated using a RNA600 Nano LabChip kit with the Agilent Bioanalyzer system (Agilent Biotechnologies) and the concentration was measured with a Nanodrop spectrophotometer (Thermo Scientific).

LMW RNA (50 µg) from *Vat^+^* and *Vat^−^* aphids was separated in a 15% denaturing polyacrylamide gel. Size fractions of 20–24 nt were excised and the RNA was extracted from the gel slices with 0.3M NaCl, followed by ethanol precipitation. The RNA was ligated to a 26 bp 5′ adapter, size-selected on a polyacrylamide gel and ligated to a 3′ adapter. The adapter sequences and PCR primers used in sRNA library preparation are provided in File S8.

First strand cDNA synthesis was performed with the adapter ligated sRNA template using the Superscript II reverse transcriptase (Invitrogen) and a primer complementary to the 3′ adapter (S 8). The cDNA was used as a template for a low cycle PCR amplification (initial denaturation of 30 sec at 98°C, 15 cycles of 98°C for 10 sec, 60°C for 30 sec and 72°C for 15 sec, followed by final extension at 72°C for 10 min) to generate sufficient template for deep sequencing. The size fractionated cDNA of ∼100 bp was gel eluted and subjected to Illumina GAII analyzer for deep sequencing.

### Bioinformatic analysis of the sRNA transcriptome

All Illumina sequencing data was initially converted to FASTA format from FASTQ. sRNA sequences were extracted from raw reads by matching them with the first 8 nucleotides of the 3′ adapter sequences. Based on the length of the mature miRNA and adapter length, sequences shorter than 18 nt and greater than 30 nt in length were removed. At this stage, the data were screened for redundant sequences. The remaining sequences were queried against ribosomal and transfer RNAs from Flybase (http://flybase.org/) and the matches to the rRNA and tRNA were discarded. Sequences having ten or more counts were assigned as unique sequences. These sequences were then queried against *A. gossypii* coding RNA regions (EST database) for perfect matches. Those sequences that matched to the sense strand were discarded as they were considered to be degraded mRNA; however those matching to the anti-sense strand were retained and aligned against the miRBase (http://microrna.sanger.ac.uk/sequences) to identify the conserved miRNA (two mismatches allowed). All bioinformatic analyses were performed using custom written PERL script. Bowtie, which is an ultra fast memory efficient short read aligner [58], was used to match the sequences to *A. gossypii* EST, *M. persicae* EST, and *A. pisum* genome sequence databases. Unaligned sequences were potential candidates for novel *A. gossypii* miRNAs. Novel miRNAs were identified using the algorithm miRDeep [22]. Since the complete genome sequence of *A. gossypii* was not available during the processing of the dataset, the EST database was used to find novel miRNAs. The bioinformatic analysis is summarized in File S1.

### *In vitro* feeding assay

A sterilized 25 ml glass beaker of 4.5 cm depth and 2.5 cm diameter was used for the *in vitro* feeding assay. At the bottom of the beaker a blotting paper disc of 2.5 cm was placed, on top of which ten aphids of same developmental stages where gently placed with a brush. Diet sachet containing [γ^-32^P]-ATP labeled ds-21 nt in 75 µl of 0.5 M sterile sucrose solution was sandwiched between two parafilm layers and this diet sachet was used to seal the mouth of the beaker. Aphids were allowed to feed on this labeled diet for 4 days. The same system, minus the [γ^-32^P]-ATP labeled ds-21 nt was used for control assay. After the end of 4 days, the number of live aphids were counted and used for RNA extraction with TRIzol® reagent. The blotting disc at the bottom of the beaker was carefully retrieved and exposed to a phosphor-imager screen and viewed in TyphoonTrio (GE Healthcare). Honeydew from these blotting discs was recovered by washing with using 0.1M Tris. The RNA extractions along with the honeydew elutions and a positive marker (labeled ds-21) was separated on a 15% PAGE, and exposed to X-ray film.

### Expression profiling of novel *A. gossypii* miRNAs by stem loop qPCR

LMW-enriched RNA was used to study the differential expression pattern of novel miRNA in the *Vat^+^* and *Vat^−^* aphid tissues. The miRNA expression was measured using a two-step process. In the first step, a stem-loop (RT) primer designed according to Chen et al. (2005) [59], was hybridized to the miRNA and reverse transcribed in a pulsed RT reaction [60]. In the second step, the RT reaction product was PCR amplified using a miRNA-specific forward primer and a universal primer (File S9) in real time with SYBR green chemistry using ABI 7500. The *A. gossypii* elongation factor-1α (*eF1α*) gene (GenBank Accession EU019874.1) was used as a reference. The relative changes in miRNA expression were quantified using Pfaffl's method (2001) [61]. The data for relative quantities were converted to fold differences by logarithmic transformation to express the data as a normal distribution. The data were represented as averages of seven measurements ± standard error and the comparisons of the miRNA expression levels between *Vat^+^* and *Vat^−^* aphid were measured using student's t-test (p≤0.05).

### Analysis of the longer sRNAs

The longer sRNAs were mapped to the transposable elements of *A. pisum*. The available transposon database of *A. pisum* was downloaded, which was predicted using the REPET transposon annotation pipeline [62]. Using bowtie short reads mapper (with non-default parameters: -k 1 -p 20 -n 1 -v 2) [57], the longer (at least 25 nt long) sequences from the two separate *Vat^+^* and *Vat^−^* aphid libraries were mapped. The sequence composition of the longer sRNA was analyzed using the DREME program [25] in the MEME motif finding software package (version 4.8.1) [26].

### Target prediction analysis

In the absence of a publicly available whole genome annotation for *A. gossypii*, several databases were used to predict the targets of the sequenced *A. gossypii* miRNAs. The first database is an extensive collection of *A. gossypii* ESTs [63], mRNAs, and gene fragments from the NCBI GenBank that were used in the construction of the *A. gossypii* Unigene build #1 (ftp://ftp.ncbi.nih.gov/repository/UniGene/Aphis_gossypii/Ago.info). From the aphid genome database resource [64] the assembled transcriptome contigs of *A. gossypii* and *M. persicae*, and the annotated gene model predictions of *A. pisum* were obtained. Supplementary file 3 (File S3) shows the number of sequences retrieved from each database. The BEDtools program [65] was used to extract the 3′-UTR sequences of *A. pisum*.

PITA and RNA hybrid programs were used for target prediction of the newly identified miRNA sequences from *A. gossypii*. As a prelude to target prediction, the PITA program was run using the default settings; a moderately stringent setting for seed region base pairing was used [30]. The moderate stringency setting allows the use of seed lengths ranging from 6–8 bases and up to single mismatch or wobble in the 7–8 nt seeds. The results of the computational prediction produced excessive number of target mRNAs for all the sequencing datasets used. To improve on the efficacy of the target predictions, the non-default parameter settings (-l 7–8 -gu “7;0,8;0” -m “7;0,8;0”) were used. This stringent setting used perfectly matching seed length of 7–8 bases. Using the recommended miRNA-target prediction energetic score (ΔΔG) cut-off for the PITA program, only targets with ΔΔG scores of −10.0 or less were retained (File S4). The RNA hybrid program was run with non-default parameters: -m 100000 -s 3utr_fly -p 0.01) to obtain a conservative set of microRNA target predictions with high statistical significance (p-value≤0.01) [33].

The BLASTX program from NCBI (blast.ncbi.nlm.nih.gov/Blast.cgi) was used (e-value cut-off used was 1.0E−08) to identify *A. gossypii* gene homologs of *A. pisum* [66]. Protein homology information was used to extract *A. pisum* GO terms associated with the *A. gossypii* sequences. The GO annotation for each dataset was imported in to the BLAST2GO web server [67]. For annotation, the GO Slim GO term mapper module in BLAST2GO was used and the generic GO term classification dataset was selected to define the functional categories of the predicted genes.

## Supporting Information

File S1 **Bioinformatic analytic scheme of sRNA libraries.**
(DOCX)

File S2 **Number of reads in each count category in both** ***Vat^+^*** **and** ***Vat^−^*** **aphid library.**
(XLSX)

File S3 **List of species and the number of sequences used in the microRNA target site predictions.**
(DOCX)

File S4 **Complete list of targets predicted for all the miRNAs identified from** ***A.gossypii*** **with ΔΔG cut-off threshold of −10.0 using PITA program.**
(XLS)

File S5 **List of targets from each database (PITA target summary).**
(XLS)

File S6 **Functional categories of the predicted target genes of the miRNAs identified from** ***A. gossypii*** **by BLAST2GO analysis using all the databases listed in File S3.**
(ZIP)

File S7 **GO term of targets predicted for all the miRNAs identified from** ***A. gossypii*** **with RNA hybrid program.**
(XLSX)

File S8 **Adapter and primer sequences in sRNA cloning.**
(DOCX)

File S9 **qPCR Primers.**
(DOCX)
