# Supplementary material for: Expression of Small RNA in Aphis gossypii and Its Potential Role in the Resistance Interaction with Melon
Source: PLoS One. 2012 Nov 16;7(11):e48579. doi: 10.1371/journal.pone.0048579 (PMC3500242; doi:10.1371/journal.pone.0048579)
Supplement: File S1 — Bioinformatic analytic scheme of sRNA libraries. (DOCX) [file pone.0048579.s001.docx]

S1. Bioinformatic analytic scheme of sRNA libraries

Raw sRNA sequences (40nt long)

Remove adaptor sequences

Filtering raw reads based on quality

Remove non-coding rRNAs and tRNA (flyBASE)

Remove duplicate sequences

sRNA sequence library with >=10 count in at least on sample

Compare to *A.gossypii* RNA with perfect match

Discard matched sense strands

Compare to miRBASE and *A.pisum* miRNA with up to 2 mismatches

Matched sequences

Unmatched sequences

miRDeep

Bowtie

Conserved miRNAs

Match to *A.gossypii* EST

Match to *A.pisum* genome sequences

Match to *M.persicae* 454 sequences

Putative novel miRNA

Target prediction

Target prediction
