# Supplementary material for: Expression of Small RNA in Aphis gossypii and Its Potential Role in the Resistance Interaction with Melon
Source: PLoS One. 2012 Nov 16;7(11):e48579. doi: 10.1371/journal.pone.0048579 (PMC3500242; doi:10.1371/journal.pone.0048579)
Supplement: File S3 — List of species and the number of sequences used in the microRNA target site predictions. (DOCX) [file pone.0048579.s003.docx]

S3. List of species and the number of sequences used in the microRNA target site predictions.

Organism Sequence type Number of sequences

*A. gossypii* ESTs 67,855

*A. gossypii* Unigenes 7,467

*A. gossypii* Contigs 3,726

*A. pisum* 3’-UTRs 36,986

*D. melanogaster* 3’-UTRs 22,908

*M. persicae* Contigs 9,771
