# Supplementary figures and images for: Expression of Small RNA in Aphis gossypii and Its Potential Role in the Resistance Interaction with Melon
Source: PLoS One. 2012 Nov 16;7(11):e48579. doi: 10.1371/journal.pone.0048579 (PMC3500242; doi:10.1371/journal.pone.0048579)

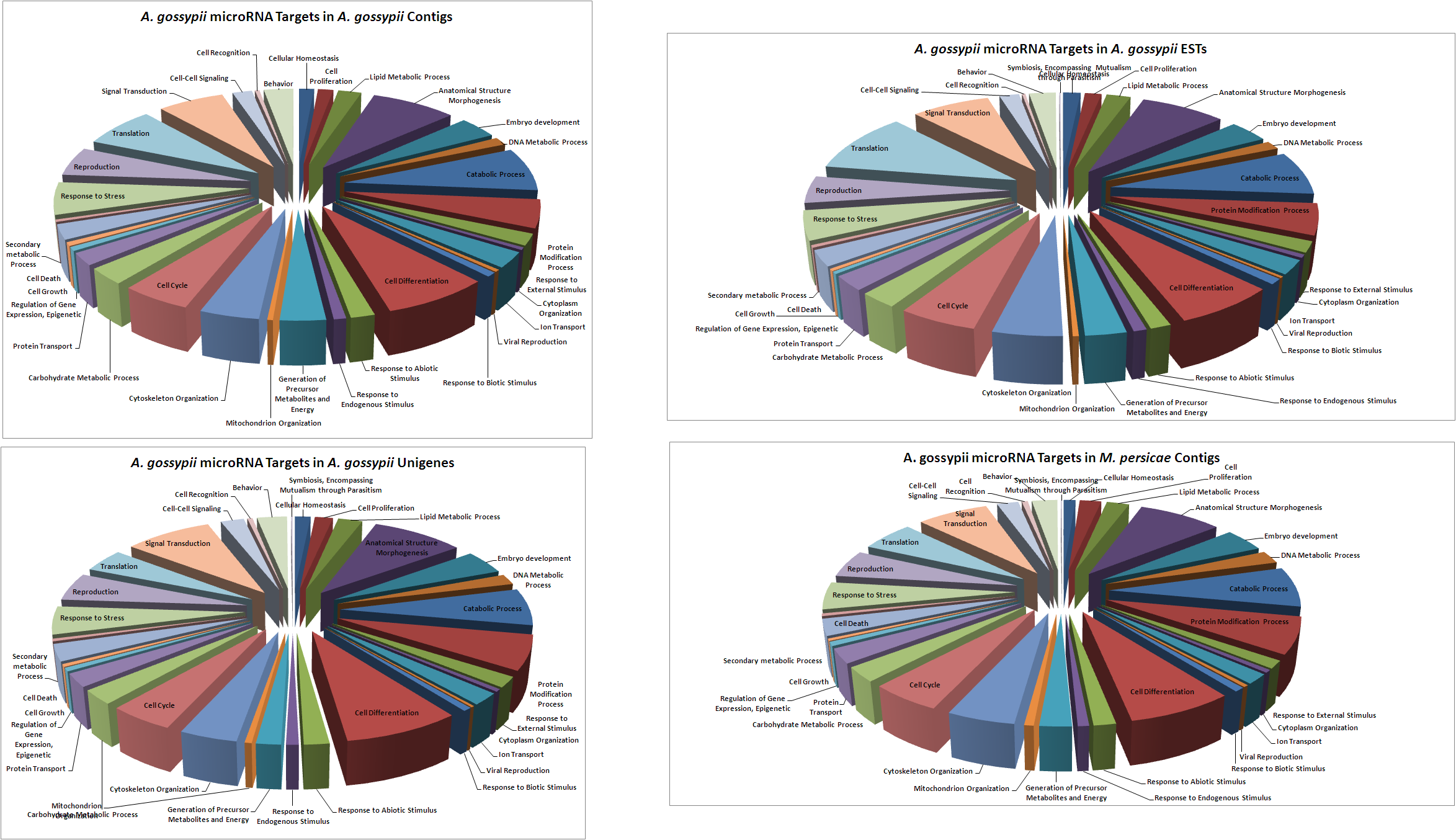

Supplement: File S6 — Functional categories of the predicted target genes of the miRNAs identified from A. gossypii by BLAST2GO analysis using all the databases listed in File S3. (ZIP) [file pone.0048579.s006.zip › S6.tif]
