# Supplementary material for: Expression of Small RNA in Aphis gossypii and Its Potential Role in the Resistance Interaction with Melon
Source: PLoS One. 2012 Nov 16;7(11):e48579. doi: 10.1371/journal.pone.0048579 (PMC3500242; doi:10.1371/journal.pone.0048579)
Supplement: File S8 — Adapter and primer sequences in sRNA cloning. (DOCX) [file pone.0048579.s008.docx]

S8. Adapter and primer sequences in sRNA cloning

| Primers and Adapter | Sequences* |
| --- | --- |
| RT Primer | 5’-CAAGCAGAAGACGGCATACGA |
| 5’ RNA adapter | 5’-GUUCAGAGUUCUACAGUCCGACGAUC |
| 3’ RNA adapter | 5’ P-UCGUAUGCCGUCUUCUGCUUGUidT |
| Small RNA PCR primer1 | 5’-CAAGCAGAAGACGGCATACGA |
| Small RNA PCR primer 2 | 5’-AATGATACGGCGACCACCGACAGGTTCAGAGTTCTACAGTCCGA |
| Small RNA sequencing Primer | 5’-CGACAGGTTCAGAGTTCTACAGTCCGACGATC |

*P represents monophosphorylated 5’ end and the 3’ end is has a blocked idT to prevent circularization.
