# Supplementary material for: Expression of Small RNA in Aphis gossypii and Its Potential Role in the Resistance Interaction with Melon
Source: PLoS One. 2012 Nov 16;7(11):e48579. doi: 10.1371/journal.pone.0048579 (PMC3500242; doi:10.1371/journal.pone.0048579)
Supplement: File S9 — qPCR Primers. (DOCX) [file pone.0048579.s009.docx]

S9. qPCR Primers

| Primer | Sequences* |  |
| --- | --- | --- |
| Universal Reverse Primer | GTGCAGGGTCCGAGGT | |
| Stemloop RT Primer | GTCGTATCCAGTGCAGGGTCCGAGGTATTCGCACTGGATACGA*NNNNNN* | |
| miR156 Forward | GCGGCGG**TGACAGAAGAGAGT** |  |
| miR157 Forward | GCGGCGG**TTGACAGAAGATAGA** |  |
| miR166 Forward | TCGCT**TCGGACCAGGCTTCA** |  |
| miR168 Forward | GCGGCGG**TCGCTTGGTGCAGGT** |  |
| miR2911 Forward | ATATATATA**GGCCGGGGACGGG** |  |
| miR2916 Forward | ATCATACC**TGGGGACTCGAAGACGA** |  |
| Ago-miRC1 Forward | ATATAA**GTCCACGGCGGTCGTC** |  |
| Ago-miRC2 Forward | TAT ATA TA**G** **GGC GGT CCG GCC GCC GCG C** |  |
| Ago-miRC3 Forward | AACTTA**GTCCACGGCGGTCGTCGAG** |  |
| Ago-miRC4 Forward | CTG ATA T**AC AAC CTC TGG CGG TC** |  |
| Ago-miRC5 Forward | GCGGCGG**AAATTCGGTTCTAGAGAGG** |  |
| Ago-miRC6 Forward | TCT GCT A**CU CGG AGT GTT AGT T** |  |
| Ago-miRC7 Forward | ATATAAT**CAAGTC GGTGTGGCG** |  |
| Ago-miRC8 Forward | ATATAAT**GGACGTATTGTCGGC** |  |
| Ago-miRC9 Forward | TATAATA**CGTCGTCCCGTCGCGT** |  |

*The underlined and italized sequence represents the six nucleotides of the 3’ end of each miRNA. The bold represents the first 13-14 nucleotides from the 5’ end of the miRNA.
